# Current stress minimization for isolated dual active bridge DC–DC converter

**DOI:** 10.1038/s41598-022-21359-1

**Published:** 2022-10-10

**Authors:** Ahmed Rashwan, Ahmed I. M. Ali, Tomonobu Senjyu

**Affiliations:** 1 Aswan University, Aswan, Egypt; 2 South Valley University, Qena, Egypt; 3 Ryukyus University, Okinawa, Japan

**Keywords:** Solar energy, Wind energy

## Abstract

This paper presents a new phase-shift modulation for isolated dual active bridge (DAB) direct current–direct current (DC–DC) converter. The proposed technique aims to minimize the maximum current stress of the converter, which could directly increase the efficiency and reduce the device losses. This modulation technique controls the converter power through only two phase-shift angles or two degrees of freedom; one phase shift is used between the legs of its first bridge and the other one between the legs of the second bridge. Although the traditional single-phase shift (SPS) technique has only one degree of freedom, it suffers from many drawbacks in terms of high current stress and reverse circulating power flow, which decrease the converter efficiency. On the other hand, increasing the number of phase-shift angles can enhance the system performance but also increase the control complexity. Thus, a comparative analysis between the proposed modulation technique and the traditional SPS was conducted; the new method showed better performance in terms of current stress reduction, along with implementation simplicity.

## Introduction

Bidirectional isolated DC–DC converters are presently the key component of many high-power devices, such as photovoltaic systems^1^, energy storages^2–4^, and electrical vehicles^4–6^. These applications require a lightweight and small power converter with high-efficiency to increase the power density; besides, galvanic isolation is needed for safety reasons. Substituting line frequency transformers with high-frequency ones led to dramatic advances in recent power converters in terms of device size, weight, and costs^7^. Among all the DC–DC converters, the dual active bridge (DAB) type is superior due to its many advantages: it provides a bidirectional power flow by simply changing the phase-shift angle between the voltage of the two bridges; the symmetric layout simplifies its dynamic modeling; zero-voltage switching is also possible for every power device without any additional circuit or special control technique, in addition to the benefit of transformer leakage inductance^8,9^. The DAB converter power can be increased by building multiport configurations and modularity topologies, which can be used as an intermediate stage in the medium-voltage power conversion system^10^.

There are many control techniques for this converter type; they are based on phase-shift control. Single-phase-shift (SPS) control is the most widely used method due to its simplicity^11,12^. Two square voltages are generated in both bridges by controlling the turning ON of the cross-connected switch pair in each bridge. Only one phase-shift angle must be adjusted between these two voltages; the power magnitude and direction can be controlled through this angle. However, reverse circulating power is caused in high current stress on the power converter. Thus, losses of the power device and magnetics components are high, weakening the converter efficiency^8^. Many attempts have been made to increase the performance of this technique. A variable duty ratio has been proposed in Ref.^13^ by calculating the phase angle value on line according to the converter’s dynamics. Some studies focused on increasing the soft-switching range^14^ or decreasing the reactive power of the converter^15^. The extended-phase-shift (EPS) control technique has been developed in Ref.^16^ to achieve better performance. It uses two degrees of freedom (i.e., inner and outer phase angles); one phase shift (the inner phase angle) controls the shift in the primary bridge diagonal switches while the other acts as in the SPS technique, that is, it controls the phase shift between the primary and the secondary bridge cross switches. The EPS control technique has dramatically decreased the reverse power and minimized the current stress in DAB converters, as well as expanded the transmission power regulating range. Nonetheless, to exchange the power direction flow, this method requires to exchange the operating states of the two bridges. The dual-phase-shift (DPS) control technique was introduced^17^ to eliminate the reactive power and increase the converter efficiency. This method uses two degrees of freedom as the EPS one but is slightly different since the inner phase-shift angle is utilized in both bridges and not only in the primary one, in addition to the outer phase shift. Extended research was also conducted to increase the DAB efficiency via triple-phase-shift control (TPS) in Ref.^18^, where three degrees of freedom are used. Other studies proposed combined and tunable phase shifts^19^, and unified phase shift control techniques^18^. However, although these methods increase the converter performance, they result in complex control and mathematical analysis as well.

This paper introduces a new phase-shift modulation technique that uses only two degrees of freedom, which enable the phase shift between the primary and secondary voltages. The first and the second phase-shift angles are between, respectively, the primary and the secondary bridge legs. With this technique, the maximum peak current is independent of the inductance between the two bridges. In this case, the peak voltage through the inductance is equal to the primary or secondary voltage and not their sum, unlike in previous techniques. Besides reducing the peak current, this approach expands the transmission power regulating range and enhances the regulating flexibility. The waveforms and operation modes of this new method introduced here. A comparative analysis involving the existing techniques was conducted. Both simulations and experimental tests were performed to verify the effectiveness of the proposed technique.

## DAB DC–DC converter

**Figure 1:**
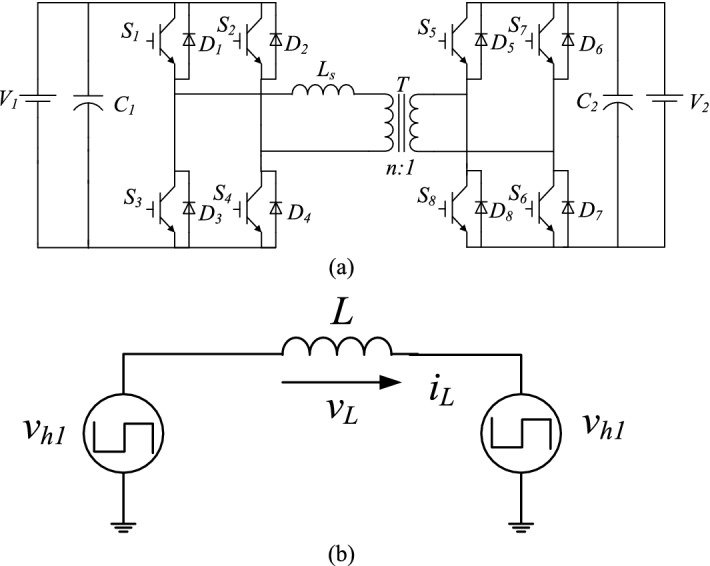
Dual active bridge (**a**) Circuit diagram. (**b**) The equivalent circuit.

**Figure 2:**
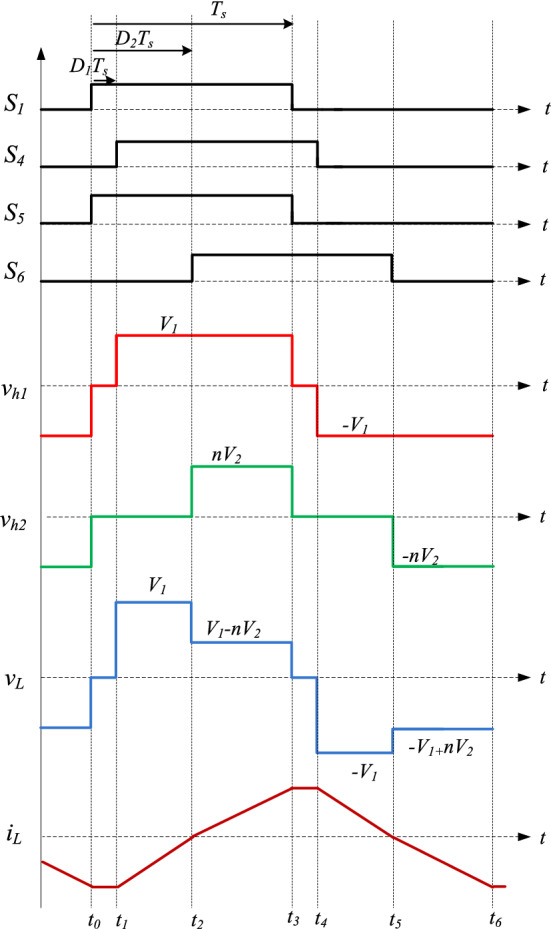
The waveform of the proposed phase shift control of the DAB converter.

Figure 1a schematizes the circuit diagram of a bidirectional DC–DC converter, which is composed of two bridges, a primary and a secondary one. The bridges are linked with the high-frequency transformer at a ratio of n:1 and an auxiliary inductor with inductance L_s_. The first bridge has two legs, each one having two switches (S_1_ and S_3_ for leg 1 and S_2_ and S_4_ for leg 2). The secondary bridge presents the same combination of four switches (in this case, labeled S_5_–S_8_). The primary bridge converts the DC input voltage (V_1_) into a high-frequency square alternating current (AC) voltage by controlling the switches S_1_–S_4_; the secondary bridge converts this high-frequency square AC voltage into a DC output voltage (V_2_) by controlling the switches S_5_–S_8_. The power flow from the primary to the secondary bridge can be controlled by the phase shift between the two AC square voltages. Figure 2b shows the equivalent circuit of the DAB converter. If the magnetizing inductance of the transformer is assumed to be greater than the leakage inductance, it can be considered as an open circuit. Thus, the DAB converter can be simply represented by two square AC voltages (V_h1_ and V_h2_) linked through inductance L (which is the sum of L_s_ and the transformer leakage inductance). The power flow direction and magnitude are controlled by adjusting the phase shift between V_h1_ and V_h2_; T_s_ is the half switching period. In this study, the power flow considered was from V_1_ to V_2_ to analyze the main operations of the proposed technique.

## Operation principles of the proposed phase shift control

### Proposed phase shift control

Figure 1a illustrates the circuit diagram of the DAB converter. In the proposed technique, one phase-shift angle (D_1_T_s_) is done between S_1_ and S_4_. A three-level voltage is synthesized on the primary side V_h1_ of the converter, unlike the traditional two-level voltage adopted in the conventional SPS control technique. This three-level voltage contributes to reducing the reverse back power to V_1_. Another phase-shift angle (D_2_T_s_) is done in the second bridge between S_5_ and S_6_; this one controls the amount of power transferred in the converter by creating an essential phase shift between the square voltages of the two bridges. The change in the D_2_ phase shift expands the transmission power regulating range, increasing the regulating flexibility. Hence, D_1_ is the phase-shift ratio between the driving gate signals S_1_ and S_4_ in the primary bridge and $0\le {D}_{1}\le 1$, while D_2_ is the ratio between the driving gate signals S_5_ and S_6_ in the secondary bridge and $0\le {D}_{2}\le 1$.

### Operation modes of the DAB converter under the proposed phase-shift modulation technique

To simplify the analysis of the bidirectional DAB converter, the device was considered under steady-state conditions. The converter can be modeled as follows (Fig. 1b): the value of the secondary bridge voltage is referred to the primary one. And ${\mathrm{V}}_{1}={\mathrm{knV}}_{2}$ and ${\mathrm{V}}_{1}>{\mathrm{nV}}_{2}$, where k is the voltage ratio and n is the transformer turns ratio. In order to simplify the performance analysis of the proposed converter, the following assumptions are made.

1. All of the power devices are ideal. The on-resistance and parasitic capacitances of the power switches are ignored, and the forward voltage drops of the diodes are neglected.
2. The leakage inductances of the couple transformer are much smaller than the magnetizing inductances, and, therefore, they neglected.

As shown in Fig. 2, the switching cycle of the converter can be divided into 6 operation modes as follow:

1. Mode 1 (t_0_ − t_1_)

**Figure 3:**
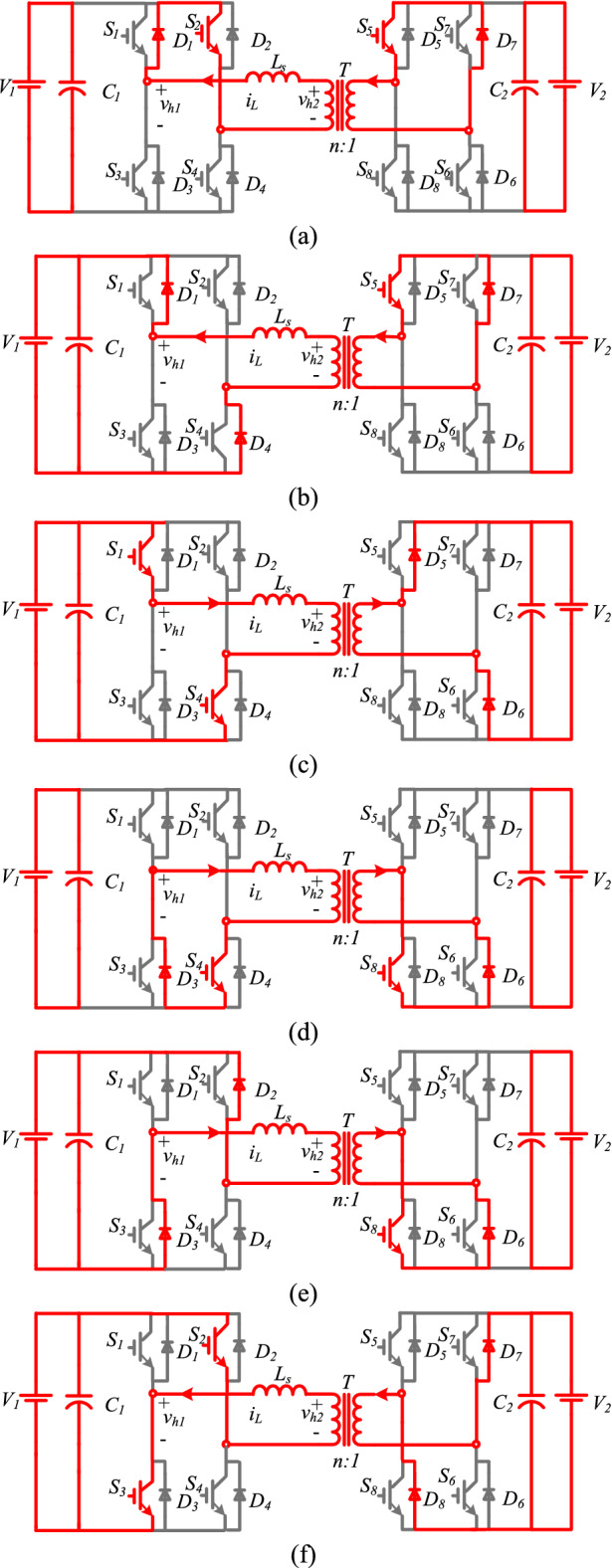
The operation modes of the DAB converter.

As shown in Fig. 3a, the inductor current ${\mathrm{i}}_{\mathrm{L}}$ is in the negative direction. At t_0_*,* the S_1_ and S_2_ are turned ON in the primary bridge, and S_5_ and S_7_ are turned ON in the secondary one. According to the current direction, the current flows through S_2_ and D_1_ in the primary bridge and through S_5_ and D_7_ in the secondary one. V_h1_ and V_h2_ are zero at this moment; thus, the voltage through L becomes zero and a constant current flows through the inductor at ${\mathrm{i}}_{\mathrm{L}}={\mathrm{i}}_{\mathrm{L}0}$.

(2) Mode 2 (t_1_ − t_2_)

Figure 3b displays the equivalent circuit of mode 2. The current is still in the negative direction. S_1_, S_4_, S_5_, and S_7_ are turned ON. According to the current direction, the current flows through D_1_ and D_4_ in the primary bridge and through S_5_ and D_7_ in the secondary one. V_h1_ is clamped to V_1_ while V_h2_ is still zero; therefore, the voltage through L is clamped to V_1_. In this mode, the current decreases linearly and can be expressed as:

1 $${i}_{L}\left(t\right)={i}_{L1}+\frac{kn{V}_{2}}{L}\left(t-{t}_{1}\right).$$

(3) Mode 3 (t_2_ − t_3_)

Figure 3c shows the equivalent circuit of mode 3. The current polarity changes from negative to positive. In this mode, S_1_ and S_4_ are still turned ON and S_5_ and S_6_ are turned ON. According to the current direction, the current flows through S_1_ and S_4_ in the primary bridge and through D_5_ and D_6_ in the secondary one. V_h1_ is still at V_1_ while V_h2_ is clamped to nV_2_. Hence, the voltage through L is clamped to ${\mathrm{V}}_{1}-{\mathrm{nV}}_{2}$. The current in this mode increases linearly and can be expressed as:

2 $${i}_{L}\left(t\right)={i}_{L2}+\frac{{V}_{1}-n{V}_{2}}{L}\left(t-{t}_{2}\right).$$

(4) Mode 4 (t_3_ − t_4_)

Figure 3d illustrates the equivalent circuit of mode 4. As shown from the waveforms in Fig. 2, mode 4 is similar to mode 1; ${\mathrm{i}}_{\mathrm{L}}$ is in the positive direction. At t_3_, S_3_ and S_4_ are turned ON while S_8_ and S_6_ are turned ON. According to the current direction, the current flows through S_4_ and D_3_ in the primary bridge and through S_8_ and D_6_ in the secondary one. Since V_h1_ and V_h2_ are zero, the voltage through L becomes zero and the current is fixed at ${\mathrm{i}}_{\mathrm{L}}={\mathrm{i}}_{\mathrm{L}3}$.

(5) Mode 5 (t_4_ − t_5_)

Figure 3e displays the equivalent circuit of mode 5. The current is still in the positive direction. S_2_ and S_3_ are turned ON while the switches S_6_ and S_8_ are turned ON. According to the current direction, the current flows through D_2_ and D_3_ in the primary bridge and through S_8_ and D_6_ in the secondary one. V_h1_ is clamped to − V_1_ while V_h2_ is still zero; thus, the voltage through L is clamped to − V_1_. The current decreases linearly and can be expressed as:

3 $${i}_{L}\left(t\right)={i}_{L4}+\frac{-kn{V}_{2}}{L}\left(t-{t}_{4}\right).$$

(6) Mode 6 (t_5_ − t_6_)

Figure 3f shows the equivalent circuit of mode 6. The current polarity changes from positive to negative. S_2_ and S_3_ are still turned ON and the switches S_7_ and S_8_ are turned ON. According to the current direction, the current flows through S_2_ and S_3_ in the primary bridge and through D_7_ and D_8_ in the secondary one. V_h1_ is still at − V_1_ while V_h2_ is clamped to − nV_2_. Therefore, the voltage through L is clamped to ${-\mathrm{V}}_{1}+{\mathrm{nV}}_{2}$. The current increases linearly and can be expressed as:

4 $${i}_{L}\left(t\right)={i}_{L5}+\frac{{-V}_{1}+n{V}_{2}}{L}\left(t-{t}_{5}\right).$$

## Transmission power and the maximum current in the DAB converter

From the equivalent circuit of the DAB converter in Fig. 1b, i_L_ can be derived as:

5 $$\frac{d{i}_{L}}{dt}=\frac{{v}_{h1}\left(t\right)-{v}_{h2}(t)}{L},$$

and according to the analysis in “Operation principles of the proposed phase shift control”, if the initial time of one switching cycle is t_0_ = 0. Then, we have t_1_ = D_1_T_s_, t_2_ = D_2_T_s_, and t_3_ = T_s_ for the positive half cycle of the input voltage. The average inductor current in the steady state over one switching period (2T_s_) is zero. Based on the waveforms in Fig. 2, the peak inductor current is equal to i_L0_ and can be expressed as:

6 $${i}_{L0}=\frac{-n{V}_{2}}{4{f}_{s}L}\left[k\left(1-{D}_{1}\right)+\left({D}_{2}-1\right)\right],$$

where ${\mathrm{f}}_{\mathrm{s}}=1/2{\mathrm{T}}_{\mathrm{s}}$ is the switching frequency and $\mathrm{k}={\mathrm{V}}_{1}/{\mathrm{nV}}_{2}$ is the voltage conversion ratio. If the power flows from V_1_ to V_2_, $\mathrm{k}\ge 1$. The current stress under the proposed method is

7 $$\left|{i}_{L0}\right|=\frac{n{V}_{2}}{4{f}_{s}L}\left[k\left(1-{D}_{1}\right)+\left({D}_{2}-1\right)\right].$$

The average transmission power of the DAB converter under the proposed phase-shift modulation can be calculated as

$$P=\frac{1}{{T}_{s}}{\int }_{0}^{{T}_{s}}{v}_{h1}{i}_{L}\left(t\right)dt$$

8 $$=\frac{n{V}_{1}{V}_{2}}{2{f}_{s}L}\left[{D}_{1}\left(1-{D}_{1}-0.5{D}_{2}\right)\right].$$

Compared with the traditional SPS control method, the current stress of the DAB converter is expressed as

9 $${i}_{max}^{c}=\frac{n{V}_{2}}{4{f}_{s}L}\left(2D-1+k\right).$$

For convenience, Eqs. (8) and (9) are defined as a unified current stress factor as follows:

$$F=\frac{{i}_{max}}{{I}_{n}}=\left[k\left(1-{D}_{1}\right)+\left({D}_{2}-1\right)\right],$$

10 $$\mathop F\limits^{{\prime }} =\frac{{i}_{max}^{c}}{{I}_{n}}=\left(2D-1+k\right),$$

where

$${I}_{n}=\frac{n{V}_{2}}{4{f}_{s}L}.$$

**Figure 4:**
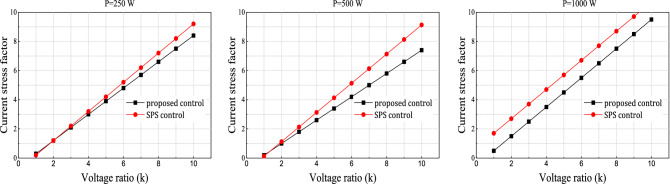
The current stress factor with the voltage conversion ratio for different power rating.

Figure 4 illustrates the relationship between current stress and voltage conversion ratio. The current stress increases along with the voltage ratio; however, the current stress factor for the proposed control technique is less than that for the traditional SPS control method for different power ratings (250, 500, and 1000 W).

**Figure 5:**
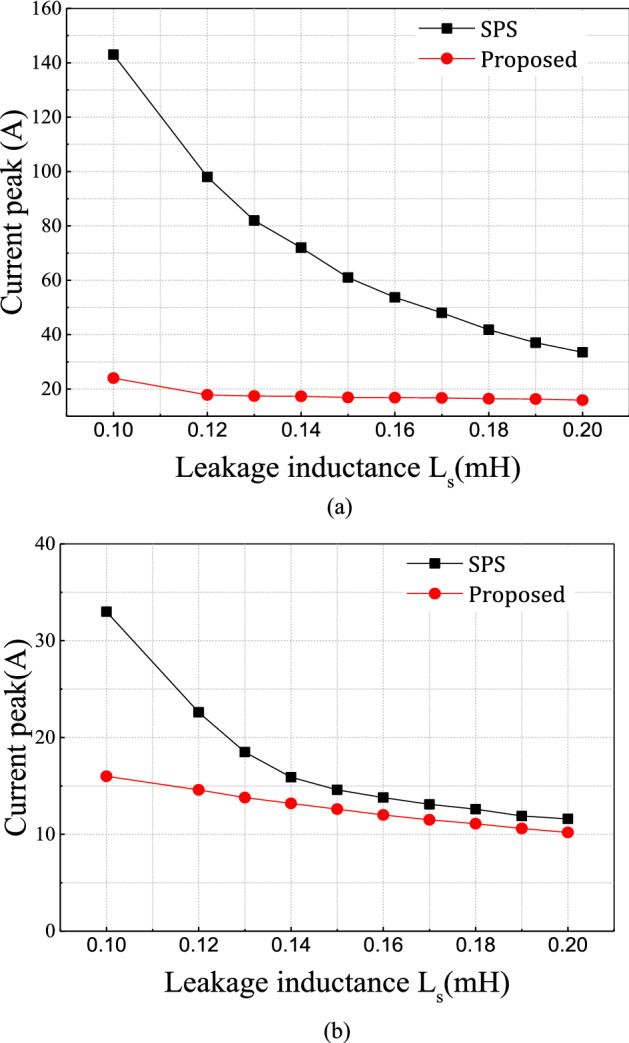
Maximum current peak varied with the inductance (**a**) switching frequency 10 kHz (**b**) switching frequency 20 kHz.

However, in the traditional SPS control method, the maximum current stress depends mainly on the leakage inductance. In SPS, the design process is challenging because it is a tradeoff between the L_s_ and f_s_ values. A smaller L_s_ increases the peak current while a larger L_s_ reduces the maximum output power in the converter. Moreover, for defined L_s_ value, the maximum current amplitude decreases when increasing the switching frequency; the switching frequency increment can be limited by the characteristics of the silicon power device. Therefore, the DAB converter should be designed carefully to work in safe operating conditions. In the proposed technique, the phase shift between the primary and secondary voltages is independent of the leakage inductance. Thus, the design process is much easier than for the SPS control method. Figure 5 compares the relation between maximum current peak and leakage inductance for the two techniques; the current peak is clearly independent of the inductance value in the proposed technique, unlike in the traditional method.

## Results and discussion

**Figure 6:**
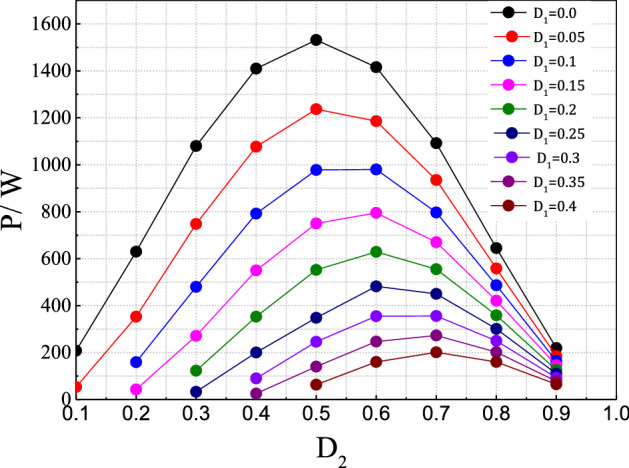
The transmission power varied with D1 and D2.

To verify the performance of the proposed control method, a DAB converter model was built (Table 1). Figure 6 shows the power regulating capacity of the DAB converter under the proposed control technique; the converter can regulate a wider range of transmission power compared to the conventional SPS control technique. The output power can be simply quantified by changing the appropriate value of the control duty ratio D_1_ or D_2_. In other words, the same amount of transmission power can be obtained by different combinations of D_1_ and D_2_, which increases the regulating flexibility. The maximum value of transmission power can be obtained when D_2_ = 0.5; the D_1_ range is 0–0.5 and D_2_ > D_1_.

**Table 1:** Main parameters of the system model.

Input DC voltage	220 V
Input DC capacitance	1500 µF
output DC capacitance	1500 µF
Auxiliary inductance	0.2 µH
Transformer voltage ratio	2
Output load	8/20 Ω
Output power	0–1.6 kW
Low Side Switches	FGH40N60SFD (600 V, 40A)
High Side switches	IRFP26N60LPBF (600 V, 26A)
Switching frequency	10 kHz

**Figure 7:**
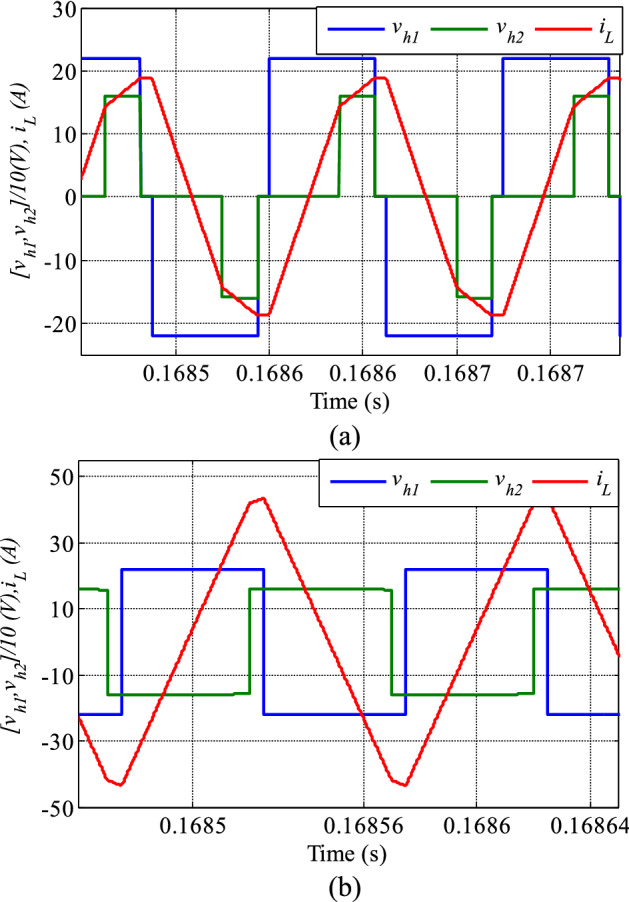
Current with voltage waveforms (**a**) proposed method (P_out_ = 800 W, D_1_ = 0.1, D_2_ = 0.7) (**b**) conventional SPS (P_out_ = 800 W, D = 0.9).

Figure 7 compares the relationship of V_h1_ and V_h2_, with i_L_ in both the conventional SPS and the proposed control methods, at the same power. V_h2_ is lagging V_h1_, which indicates that the power is flowing from the primary to the secondary side. Besides, the V_h1_ amplitude is higher than the V_h2_, revealing a step-down mode operation (k > 1). The maximum current peak in the proposed method is lower than that in the conventional SPS control technique despite both approaches transmit the same power. The benefit of current stress reduction is the decrease in the losses in the converter and the power device rating.

**Figure 8:**
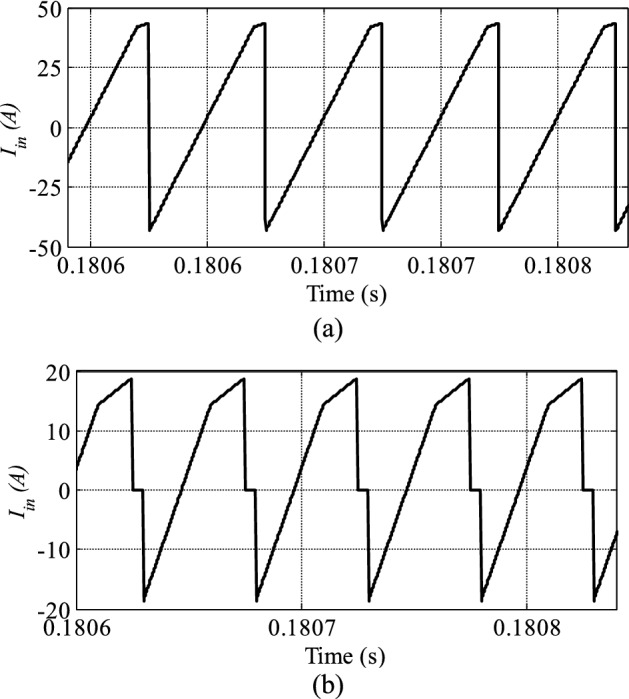
The DAB converter input current P_out_ = 800 W. (**a**) SPS control (**b**) proposed control.

Figure 8 illustrates the converter input current for both methods for the same transmission power. The reverse power in the conventional SPS control technique is higher than that in the proposed method. Decreasing the reverse power can assist in decreasing the power circulating currents between the bridges.

**Figure 9:**
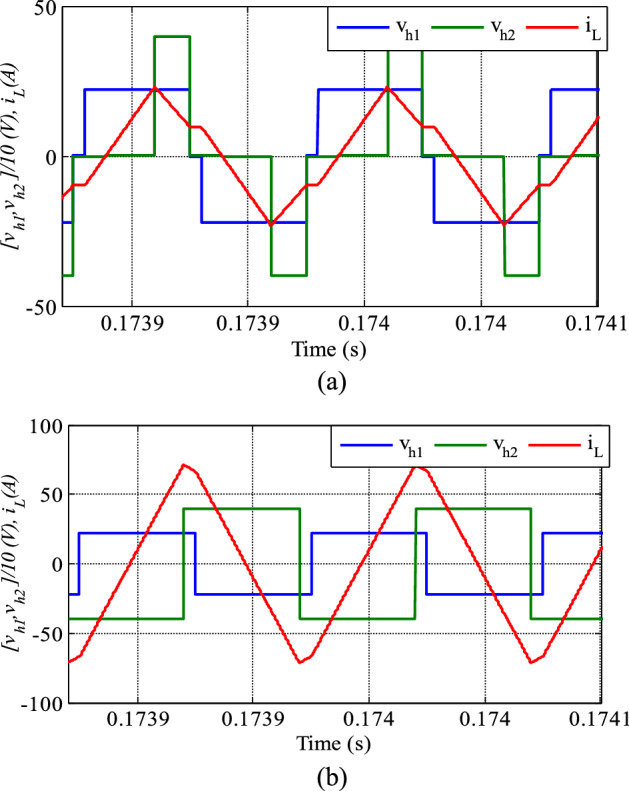
Current with voltage waveforms (**a**) proposed method (P_out_ = 2000 W, D_1_ = 0.1, D_2_ = 0.7) (**b**) conventional SPS (P_out_ = 2000 W, D = 0.9).

To verify the effectiveness of the proposed technique, the system was tested under different voltage ratios below 1. Figure 9 compares the proposed method with the traditional SPS control technique for k = 0.55, clearly showing a much lower current stress with the new method. The converter is in boost mood in this test which V_h2_ is greater than V_h1_ and the power flow direction is from V_h1_ to V_h2_.

**Figure 10:**
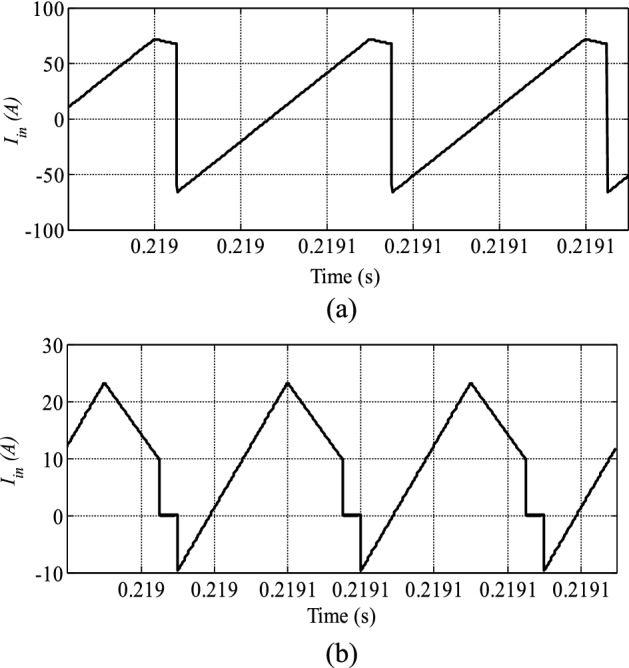
The DAB converter input current P_out_ = 2000 W. (**a**) SPS control (**b**) proposed control.

Figure 10 shows the converter input, current which has a negative value proportional to the amount of reverse power in the device. In the proposed technique, the reverse power is also lower than that in the SPS control method.

**Figure 11:**
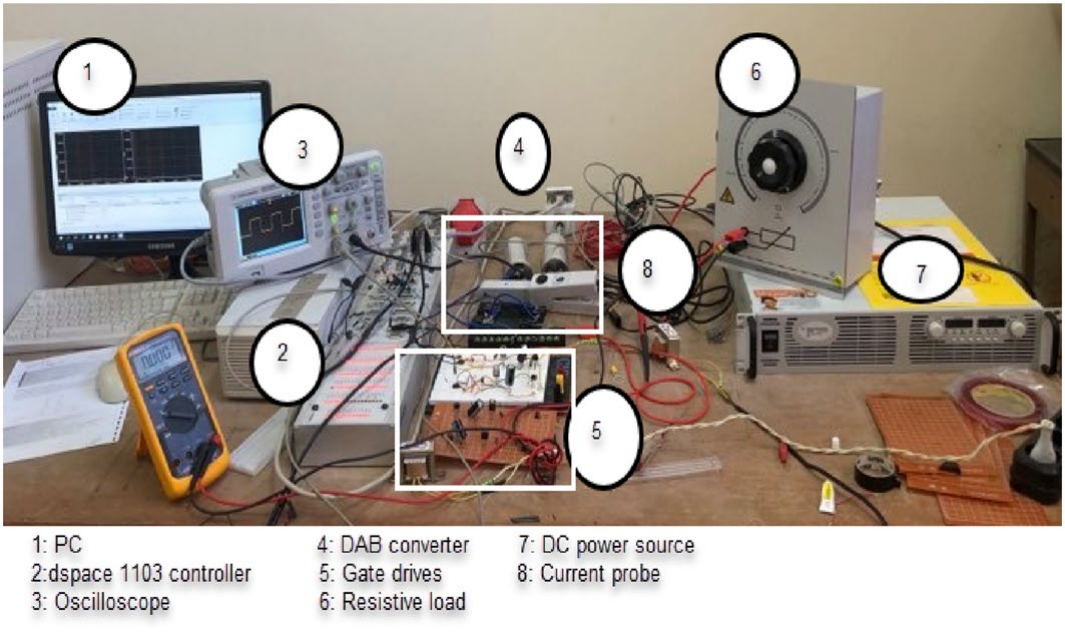
Experimental prototype system.

**Figure 12:**
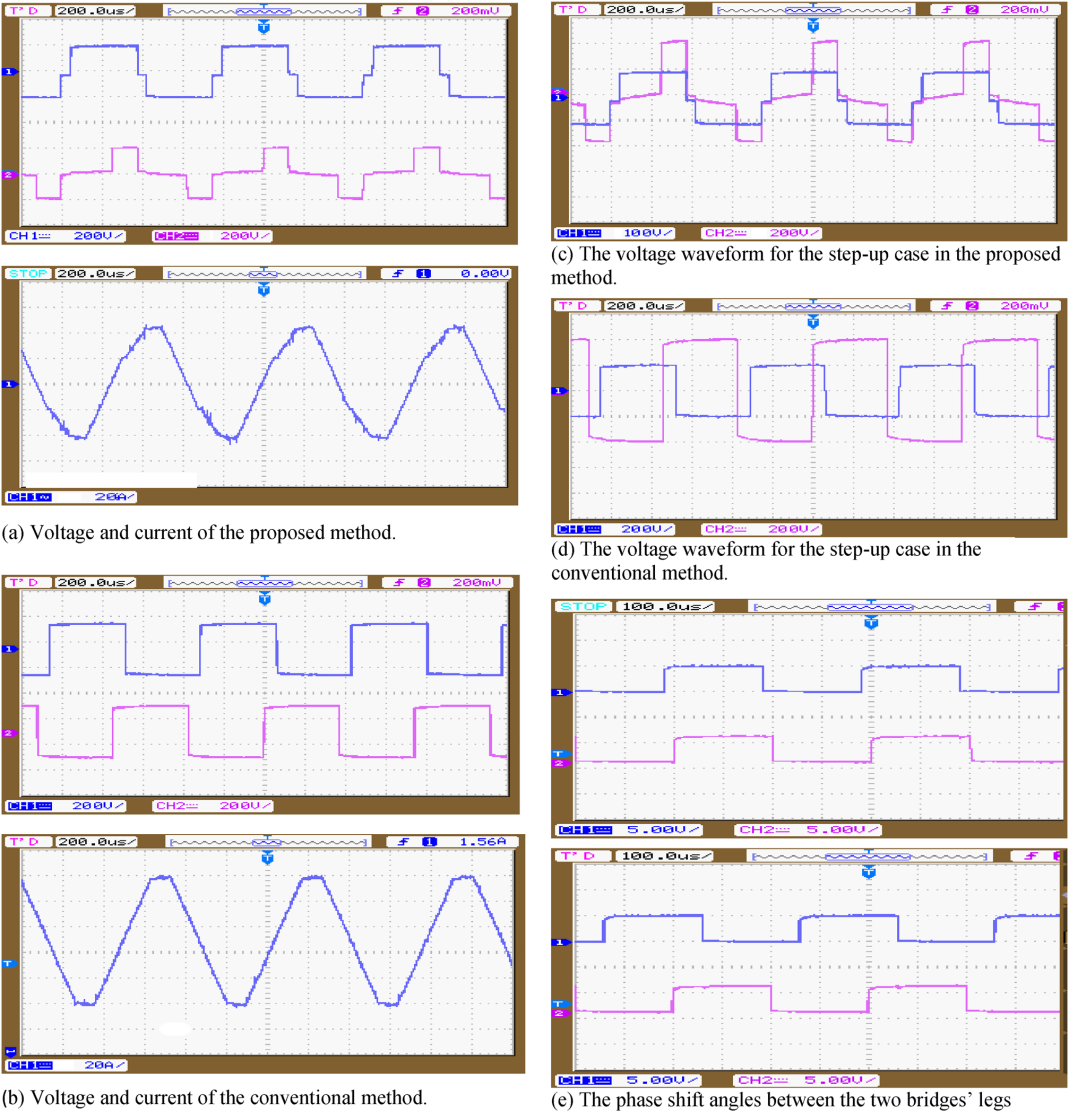
Experimental results for the current with voltage waveforms (**a**) proposed method (Pout = 800 W, D1 = 0.1, D2 = 0.7) (**b**) conventional SPS (Pout = 800 W, D = 0.9).

Experimental test has been accomplished to confirm the simulation results. A prototype DAB converter (1.6 KW) has been built to develop the output voltages and currents. Hardware and parameters of the proposed topology is presented in Table 1. The IGBT (FGH40N60SFD), with nominal voltage 600 V, nominal current 40 A is used for the first bridge, and IGBT (IRFP26N60LPBF) with nominal voltage 600 V, nominal current 26 A is used for the second bridge, DSpace DS 1103 controller is used to produce the gating signals. A photo of the experimental setup is shown in Fig. 11. Figure 12 displays the experimental results, where the voltage and current of the proposed method is shown in Fig. 12a, voltage and current of the conventional method is shown in Fig. 12b, the voltage waveform for the step-up case in the proposed method is shown in Fig. 12c, The voltage waveform for the step-up case in the conventional method is shown in Fig. 12d, and the phase shift angles between the two bridges’ legs is shown in Fig. 12e. Results confirm the veracity of the submitted analysis waveform, subsequently both simulation and experimental results shows the versatility and flexibility of the proposed DAB converter proposed control method.

Comparisons of the control techniques for DAB dc-dc converters are shown in Table 2. The switching frequency is adjusted widely because the control techniques in Refs.^13,16^ are based on SPS, EPS and TPs, respectively. There is increased conduction loss because the operating modes in Refs.^13,16,18^ are not globally ideal. It is possible to accomplish the ZVS performance in Refs.^13,18^ with the help of the auxiliary component or the transformer's magnetizing inductance, which also results in increased conduction loss. The design is more complicated and less adaptable for a broad conversion ratio since the modulation strategy in Ref.^13^ is dependent on the off-line computation. The suggested control method is capable of achieving ZVS by itself, without the use of any auxiliary parts. Without using an off-line computation, the modulation method is implemented in real-time. As a result, developing and implementing the control approach is simpler.

**Table 2:** Comparisons of control strategies for the DAB converter.

	^13^	^16^	^18^	Proposed method
Switching frequency	Variable frequency	Variable frequency	Constant frequency	Constant frequency
Control strategy	SPS	EPS	TPS	Modified DPS
Solution of the strategy	Off-line	Real time	Real time	Real time
Working modes	Not optimal	Not optimal	Locally optimal	Globally optimum
Soft switching strategy	Auxiliary component	Not global soft switching	Magnetizing inductance	Inherent
Design computationl complexity	Low	High	Moderate	Moderate
Current stress reduction	Low	Medium	Medium	High
Wide conversation ratio capacity	Low	High	Moderate	Moderate

## Conclusion

This paper proposes a new phase-shift modulation technique for the DAB converter. The new technique uses only two degrees of freedom, which enable the phase shift between the primary and secondary voltages. The waveforms and operation modes of this new method introduced here. A comparative analysis involving the existing techniques was conducted. Both simulations and experimental tests were performed to verify the effectiveness of the proposed technique. It is observed from the results that the peak current is reduced by 50% compared to the conventional phase shift modulation technique. The inverse operation of the converter is easy to operate, in addition to the flexibility of the transmission power through the system.

## Data Availability

The datasets generated during and/or analyzed during the current study are available from the corresponding author on reasonable request.
